# Sexually Selected Infanticide in a Polygynous Bat

**DOI:** 10.1371/journal.pone.0025001

**Published:** 2011-09-16

**Authors:** Mirjam Knörnschild, Katja Ueberschaer, Maria Helbig, Elisabeth K. V. Kalko

**Affiliations:** 1 Institute of Experimental Ecology, University of Ulm, Ulm, Germany; 2 Smithsonian Tropical Research Institute, Balboa, Panama; University of Western Ontario, Canada

## Abstract

**Background:**

Adult individuals of many species kill unrelated conspecific infants for several adaptive reasons ranging from predation or resource competition to the prevention of misdirected parental care. Moreover, infanticide can increase the reproductive success of the aggressor by killing the offspring of competitors and thereafter mating with the victimized females. This sexually selected infanticide predominantly occurs in polygynous species, with convincing evidence for primates, carnivores, equids, and rodents. Evidence for bats was predicted but lacking.

**Methodology/Principal Findings:**

Here we report the first case, to our knowledge, of sexually selected infanticide in a bat, the polygynous white-throated round-eared bat, *Lophostoma silvicolum*. Behavioral studies in a free-living population revealed that an adult male repeatedly attacked and injured the pups of two females belonging to his harem, ultimately causing the death of one pup. The infanticidal male subsequently mated with the mother of the victimized pup and this copulation occurred earlier than any other in his harem.

**Conclusions/Significance:**

Our findings indicate that sexually selected infanticide is more widespread than previously thought, adding bats as a new taxon performing this strategy. Future work on other bats, especially polygynous species in the tropics, has great potential to investigate the selective pressures influencing the evolution of sexually selected infanticide and to study how infanticide impacts reproductive strategies and social structures of different species.

## Introduction

Nonparental infanticide, *i.e.* the killing of infants by not directly related conspecifics, is a rarely-observed behavior on the species level. However, it is generally a widespread behavior in the animal kingdom [1], ranging from invertebrates to vertebrates such as birds [2] and mammals [3]. Among mammals, non-parental infanticide has been unequivocally documented in eight orders, predominantly in primates [4], rodents [5], and carnivores [6], and, to a lesser extent, in artiodactyls [7], perissodactyls [8], lagomorphs [9], scandentias [10], and chiropterans [11].

Several hypotheses, none of which are mutually exclusive, attempt to explain the occurrence of nonparental infanticide [3], [12]. The social pathology hypothesis [13], [14] implies that infanticide is a maladaptive behavior limited to recently disturbed habitats or crowded social conditions [15], [16]. However, this nonadaptive explanation of infanticide is not supported by game theory models [17]. In contrast, other hypotheses consider nonparental infanticide an adaptive behavior with clear benefits for the infanticidal individual. On the one hand, the predation hypothesis suggests that infanticide renders nutritional benefits for the aggressor [3], [12]. In rodents, infanticidal females often consume their victims, especially during the energy demanding period of lactation [18], [19]. Correspondingly, male rodents commit infanticide predominantly during periods of food deprivation [20], [21]. On the other hand, the resource competition hypothesis postulates that infanticide provides the aggressor or its descendents with improved access to limited resources such as food, shelter, or territory [3], [12]. In group-living carnivores, the dominant female regularly kills the young of subordinate females which, in turn, often help in rearing her own offspring [22], [23]. Similarly, female rodents may kill the young of females with which they compete for shelter or territory access because victimized females will leave an area after losing their litters [9], [24]. Furthermore, the adoption avoidance hypothesis implies that infanticide is committed to avoid the provisioning of unrelated young [3], [12]. The most convincing evidence for this hypothesis comes from pinnipeds; lactating females often attack alien pups that are attempting to steal milk [25], [26].

The sexual selection hypothesis predicts that infanticide is a male reproductive strategy in which infanticidal males kill the offspring of competing males in order to increase their own reproductive success [3], [12]. Sexually selected infanticide primarily occurs in species that exhibit intense male-male competition and feature short reproductive tenure of males [27]. Correspondingly, sexually selected infanticide is found mainly in polygynous mammals such as primates [4], lions [6], equids [8], [28] or murid rodents [29] but it occurs in solitary species such as brown bears as well [6], [30]. Sexually selected infanticide is most advantageous in species with a flexible female reproductive cycle that allows victimized females to conceive again soon after losing an infant [3], [12]. However, sexually selected infanticide also occurs in strictly seasonal breeders, where it shortens inter-birth intervals [31] or increases the quality of future offspring [32].

In bats, evidence for infanticide is very limited [11] which is probably caused by their secretive, nocturnal lifestyle. Female Indian false vampire bats, *Megaderma lyra* (Megadermatidae), supplement their diet by cannibalizing alien young [33], supporting the predation hypothesis as an explanation for infanticide. Female greater spear-nosed bats, *Phyllostomus hastatus* (Phyllostomidae), attack and presumably kill offspring belonging to different social groups [34], a finding probably supporting the predation and/or resource competition hypothesis. Female Mexican free-tailed bats, *Tadarida brasiliensis* (Molossidae), may attack and fatally injure alien pups that attempt to steal milk [35], which might support the adoption avoidance hypothesis. However, there was no evidence for infanticidal behaviour of male bats in general or sexually selected infanticide in particular [11]. Our study species, the white-throated round-eared bat *Lophostoma silvicolum* (formerly *Tonatia silvicola*, see [36]), is a promising species for the study of sexually selected infanticide in bats. *Lophostoma silvicolum* is a medium-sized New World leaf-nosed bat (Phyllostomidae; [37]) that uses small foraging areas in close proximity to its roost to prey mainly on large arthropods [38], [39]. For roosting *L. silvicolum* exclusively uses cavities in active termite nests, mainly of the arboreal species *Nasutitermes corniger* [40], [41]. These cavities are excavated and maintained by males only and provide shelter for one male and a small group of females and their dependent offspring, implying a mating system based on resource-defence polygyny [42]. The study by Dechmann and colleagues [42] showed that, whenever females were present in a roost, a single adult male was found in the vast majority of cases (34 cavities sampled; only one cavity contained a harem group with a dominant adult male and a subordinate subadult male), indicating that subordinate males within a harem are exceedingly rare. Males without a harem roost alone or in bachelor groups [42], [43]. A single offspring is born once or rarely twice a year per female [42]. Both male and female offspring disperse from their natal roost before reaching sexual maturity [44]. Extra-harem paternities occur frequently, with more than 50% of pups being sired by males that were not resident in the respective harems the pups were born in [42].

Several facts predicting the occurrence of sexually selected infanticide in other species [3], [12] are also found in *L. silvicolum*: there is intense male competition for females [42], male tenure in female groups can be as short as 12 months [44] and females are polyestrus [42], which makes them able to respond to the death of an infant by conceiving again as soon as possible. Here, we document infanticidal male behavior towards dependent pups under natural conditions, making *L. silvicolum* the first bat species known to exhibit sexually selected infanticide.

## Results

### Male aggression

We used behavioural observations during 13 nights throughout a 71 days period to classify social behaviors of one group of free-living *L. silvicolum* in its roosting cavity. Behaviors ranged from ubiquitous comfort behaviors such as autogrooming to sex-specific behaviors such as roost construction or maternal care (for details, see Table 1). Particularly conspicuous male-specific behaviors consisted of aggression towards non-volant pups that were left in the roost by their mothers. On 47 occasions in seven nights, we observed the adult male approaching the two pups, sniffing them, then seizing them with his wings and applying bites. During the male's approach, both pups exhibited a protective position: they hid their heads under their partly opened wings and pressed their body towards the ceiling of the roosting cavity. Before attacking, the male did not seem visibly agitated; in all 47 cases, he attacked the pups after autogrooming or roost maintenance.

**Table 1:** Ethogram describing behaviors exhibited by *L. silvicolum* in the roost.

Behavior	Description	Category	Age	Gender
Roosting *	Spending time in the roost without displaying any activity	Roosting	all	all
Scanning *	Rapid movements of ears, often with partly opened mouth	Roosting	all	all
Roost maintenance *	Excavation and maintenance of roost cavity in termite nests by tearing off nest material with teeth	Maintenance	adults	males
Belly presentation *	Presenting the belly to conspecific in the roost by stretching the closed wings back, often when entering the roost	Inspection	adults	all
Belly sniffing *	Sniffing the belly region of conspecific, often when the latter is entering the roost	Inspection	adults	all
Nose-to-nose sniffing	Sniffing the nose region of conspecific	Inspection	adults	all
Unfocussed sniffing	Sniffing towards conspecific without body contact or focus on specific body regions	Inspection	adults	all
Flehming	Curling back upper lip (it is unclear whether *L. silvicolum* possesses a vomeronasal organ)	Inspection/Mating	adults	males
Copulation attempt *	Copulating belly-to-back; initiated by both sexes	Mating	adults	all
Fight	Grappling with conspecific; often grasping opponent with both wings and biting its neck region	Aggression	adults	all
Expulsion *	Aggressively preventing conspecific from entering the roost	Aggression	adults	males
Aggression towards pups	Retaining pup with both wings and biting neck, forearm, and wings; may be followed by shaking and subsequently dropping the pup (Fig. 1)	Aggression	adults	males
Protective position	Covering the head with partly unfolded wings while pressing the body against the roost surface	Defence	pups	all
Plummeting	Loosening grip on roost surface in order to avoid aggression	Defence	pups	all
Autogrooming	Cleaning of fur and wings with the tongue and claws of hindfeet	Comfort	all	all
Yawning	Exposing gum and teeth briefly	Comfort	all	all
Defecating	Pushing body away from substrate with wrists and feet	Comfort	all	all
Allogrooming	Maternal grooming of pup	Mother-pup interaction	adults	females
Nursing	Resting on mother while being attached to the teat	Mother-pup interaction	pups	all
Solicitation	Soliciting maternal care by climbing towards the mother and seeking the teat	Mother-pup interaction	pups	all
Licking	Extensively licking the corners of the mother's mouth	Mother-pup interaction	pups	all
Pick-Up	Retrieving fallen pup	Mother-pup interaction	adults	females
Transport	Transporting non-volant pup out of danger (e.g. after predation event in the roost)	Mother-pup interaction	adults	females
Shake-Off	Rhythmic muscle contractions of the whole body as maternal signal for the pup to detach from the teat	Mother-pup interaction	adults	females
Feeding	Consuming prey items in the roost	Miscellaneous	adults	all
Climbing	Moving through roost while hanging from the hindfeet	Miscellaneous	all	all
Loosing foothold *	Falling accidentally during grooming or roost maintenance	Miscellaneous	all	all
Flight practice	Practicing flight by rapidly flapping wings while hanging from the feet	Miscellaneous	pups	all

‘Inspection’ behaviors were used during ritualized greeting ceremonies between roost mates. Behaviors marked with asterisks have already been reported in previous studies [38], [41]–[43].

**Figure 1:**
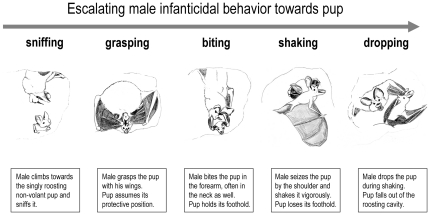
Escalating male infanticidal behavior. The sketches are depicting different male infanticidal behavior types escalating in violence. The illustrated sequence of behaviors lasted more than one minute. Sketches were made by M. Helbig from infrared video recordings. Angle of view is from below the arboreal termite nest into the roosting cavity.

The attacks varied in intensity. During mild attacks (n = 43), pups adopted their protective position but managed to not lose their foothold and stay in the roost. During heavy attacks (n = 4), pups fell out of the roost or plummeted down, probably on purpose to avoid being bitten. On one occasion, the male repeatedly and severely bit one pup in its neck, shoulder and forearm, shook it vigorously and then dropped it. The attack (Fig. 1) lasted more than one minute and seriously injured the pup. The inflicted wound was bleeding visibly and the pup still held its injured wing in an awkward angle three days after the attack.

Attacks exclusively happened when the pups' mothers were not present in the roosting cavity. In 79% of cases (n = 37) and during all four heavy attacks, the male was alone with the pups. In the remaining 21% of cases (n = 10), one or more adult females, but never the respective mothers, witnessed the attack. None of the females present interfered. Whenever the pups fell out of the roost, they had to be retrieved by their mothers once the latter returned from foraging. The injured pup fell out of the roost several days after it got wounded and was probably predated upon before its mother could retrieve it as the mother did not bring it back and we were unable to find it in close vicinity to the roost. The second, younger pup was successfully retrieved by its mother whenever it fell out of the roost and thus survived all male attacks. As genetic samples were not collected, we can only hypothesize that the dead pup was not sired by the infanticidal male.

### Copulations

In total, we observed 49 copulation attempts in five nights; we used the term ‘copulation attempt’ instead of ‘copulation’ because we had no way of investigating whether copulations were successful or not. Copulation attempts lasted seven seconds on average (range: 2–14 seconds) and could be initiated by both sexes, but male initiation was significantly more prevalent (χ^2^ = 24.083, df = 1, p<0.0001). The majority of copulation attempts (42 of 49) were initiated by the male intently sniffing the female's genital region and flehming (*i.e.*, curling back the upper lip). Copulation attempts were performed belly-to-back. We rarely witnessed aggressive encounters (all of which were considered to be mild) between the male and the females prior to or during copulation attempts. Females sometimes terminated copulation attempts by flying or climbing away (in six of 49 cases).

**Figure 2:**
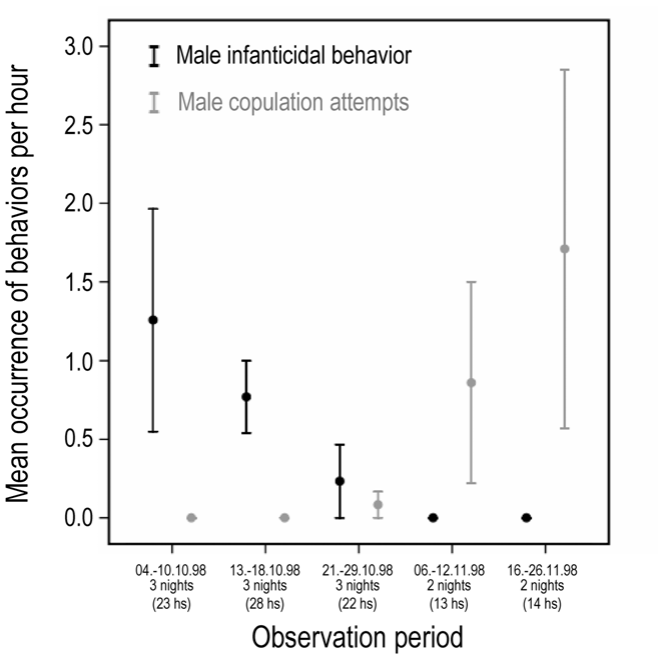
Male infanticidal behavior and subsequent copulation attempts. The frequency of occurrence of male infanticidal behavior and copulation attempts is depicted over time. Total observation time adds up to 100 hours. Means ± SE are shown.

**Figure 3:**
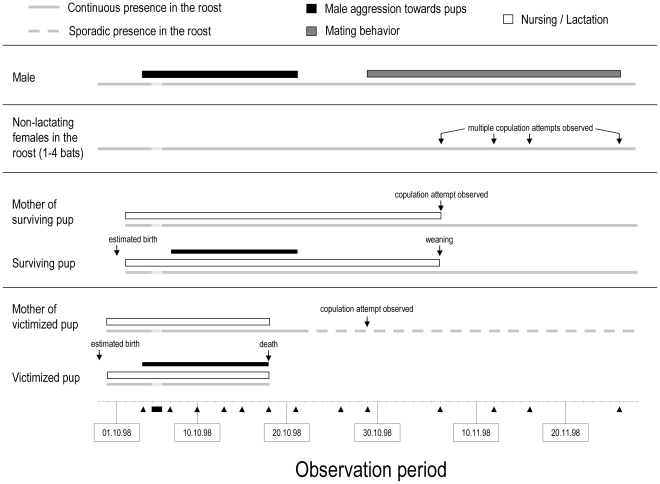
On- and off-set of social behaviors during the observation period. An overview of the timing of selected social behaviors in adult and juvenile *L. silvicolum* is shown. A visual census was conducted daily. Black triangles below the timeline: dates with nightly video observations. Black bar below the timeline: the roost was unoccupied on two consecutive days (5.–6.10.1998) following an unsuccessful predation attempt by a snake. For clarity, data for those two days are extrapolated (depicted in light grey).

During our study period, the frequency of male infanticidal behavior decreased significantly (Spearman's rho: r = −0.766, n = 13, p = 0.002, α = 0.025), whereas the frequency of male copulation attempts increased significantly (Spearman's rho: r = 0.831, n = 13, p<0.001, α = 0.025; Fig. 2). The male copulated with all females in the roost, sometimes on multiple occasions. The number of adult females in the roost fluctuated daily between three to six bats; the two lactating females were present on every census day. In case of the latter, copulation attempts occurred only after the pup was either weaned or dead, respectively. The male copulated seven days earlier with the mother of the victimized pup (eleven days after the pup's death) than with the mother of the surviving pup (Fig. 3).

## Discussion

Male aggression towards pups not only caused injuries but also falls from the roosting cavity. Fallen non-volant bat pups are highly subjected to predation by snakes, ants and various nocturnal mammals [34], [45], which is why we consider the male aggression towards pups to be infanticidal. In contrast to reports from other species where infanticide often happens when baseline aggression is already raised (e.g. in the tumult of inter-group encounters in primates; [46]–[48]), the *L. silvicolum* male did not seem visibly agitated before attacks: In all 47 cases, he attacked the pups after autogrooming or roost maintenance, making it unlikely that the observed infanticidal behavior was simply the consequence of a previously raised level of aggression.

Among the four hypotheses proposed as an adaptive explanation for non-parental infanticide [3], [12], the sexual selection hypothesis best explains the observed male aggression in *L. silvicolum*. Expectations of the nutritional benefit hypothesis are not fulfilled because the victimized pup was not consumed by the aggressor. The adoption avoidance hypothesis cannot explain our observations either, because the pups were attacked exclusively by the male and not by lactating females and we never observed females reacting aggressively towards approaching alien pups. The resource competition hypothesis seems an improbable explanation for the observed male infanticidal behavior as well, since it predicts that infanticide causes an increased access to resources either for the aggressor or its offspring. While roosting space is certainly an important resource for bats in general [49]–[50] and *L. silvicolum* in particular [38], [40], [41], it is very unlikely that the male committed infanticide in order to increase the available space in his roosting cavity to accommodate more estrous females or offspring sired by him. The number of females in the roost fluctuated daily by a factor of two and individuals readily roosted in body contact with one another, which makes a limitation in available roosting space for visiting estrous females or their offspring unlikely [42]. Furthermore, previous observations demonstrated up to 20 individuals roosting together in similar-sized cavities of termite nests (EK, unpublished data).

The sexual selection hypothesis, however, is in complete concordance with our observations. Four main assumptions need to be fulfilled in order to consider infanticidal behavior to be sexually selected: infanticide must be committed exclusively by males, the infanticidal males must successively mate with the mothers of the victimized infants, the inter-birth interval of the respective mothers must be shortened by the infanticide and the victimized infants must be unrelated to the aggressors [3], [12].

In *L. silvicolum*, aggression towards pups was exclusively male-specific. The infanticidal male not only mated with the mother of the victimized pup but also started copulating with her earlier than with any other female in his roost. Regarding the relatedness between aggressor and victimized pup, two potential scenarios could result in a harem male not being related to infants born in his harem: Firstly, females impregnated by another male could join his harem [42]; secondly, the harem male could replace another male and gain access to a group of females impregnated by his predecessor. Female *L. silvicolum* have been observed to switch between harem groups throughout the year [42], [43]. On average, 46% of the pups born in a harem are fathered by the current harem male [42]. The replacement of a harem male by a competitor has not been observed yet [42], but we have strong evidence that males guard their roosting cavity vigilantly and aggressively expel competing males ([43]; own observations). In our study, either of the above mentioned scenarios could have taken place before we started our observations. We can, however, only hypothesize that the victimized pup was not sired by the infanticidal male, because genetic samples were not collected.

Given that more than 50% of *L. silvicolum* pups were sired by males outside the respective harem group [42], we conclude that, for species with sexually selected infanticide, the level of extra-group paternity varies more than previously indicated. In other species exhibiting sexually selected infanticide, extra-group paternity is low or virtually absent (lions [51], red howlers [52], chacma baboons [53], Hanuman langurs [54], chimpanzees [55]), thus facilitating paternity assessments for usurping extra-group males. In contrast to the above mentioned species, which often form multi-male multi-female groups with considerable reproductive skew between males, *L. silvicolum* almost always forms single male groups [42]. It is unknown how *L. silvicolum* males may assess paternity.

The fact that the victimized female resumed sexual receptivity earlier than other *L. silvicolum* females warrants further discussion. Polyestry is not a necessary prerequisite for sexually selected infanticide; even in monoestrus species, especially ones with prolonged gestation and lactation, infanticide can considerably shorten inter-birth intervals [6], [31] or enhance the quality of future offspring [32]. Nevertheless, sexually selected infanticide is much more likely to occur in polyestrus species [3], [12]. In our study area, female *L. silvicolum* exhibit bimodal polyestry with two reproductive peaks in March/April and August/September [42]. Even though the two peaks are distinct, a few pregnant females can be found throughout most of the year (Charles Handley, personal communication), suggesting that female reproduction is not strictly synchronized. Female bats that give birth out of synchrony with conspecifics may have altered infanticide-induced estrous cycles [11]. Bimodal polyestry was interpreted to indicate a post-partum estrus in *L. silvicolum* [42], whereas our results show that female *L. silvicolum* exhibit lactational amenorrhea and a post-lactational estrus as observed in other mammals [56], [57]. In our study, the infanticide halved the lactational amenorrhea of the victimized female and expedited her estrus. Thus, to our opinion (but see [43]), male infanticide in *L. silvicolum* has the potential to accelerate female sexual receptivity. We do not know, however, exactly how much the inter-birth interval of the victimized female in our study was shortened compared to what the inter-birth interval would have been for this female without infanticide. In other species, infanticidal males are able to shorten inter-birth intervals by half or more (eight vs. 15 months in Hanuman langurs [58], [59]; eight vs. 18 months in lions [6]). In langurs, the younger the victimized infant, the more an aggressor gains from infanticide (i.e. the shorter the subsequent inter-birth interval; [31], [60]).

Male infanticidal behavior is more frequently observed in species with short male tenure and long female lactational amenorrhea. For comparison, average male tenure is 2.2±1.6 years in langurs [54] and 3.5±2.1 years in lions [61]. A tenured male benefits by inseminating available females as soon as possible so that his offspring is weaned and thus out of danger before the male is replaced by an infanticidal successor [58]. In *L. silvicolum*, male tenure rarely exceeds 30 months and might be as short as twelve months [42], [44], thus potentially spanning only two female reproductive cycles. An individual male's tenure can be limited by the longevity of the live termite nest used as a roost (up to 30 months; [44]), and presumably by takeovers of male competitors.

Overall, the relatively short male tenure may have been a prerequisite for the evolution of sexually selected infanticidal behaviour in *L. silvicolum*. This raises the question how common sexually selected infanticide in *L. silvicolum* is. In our study, we observed male infanticidal behavior in more than half of our observation nights (seven out of 13), whereas an earlier study found no evidence for male infanticidal behavior in eleven full-night observations of one group [42]. The observations by Dechmann and colleagues [42] were dispersed over time in order to cover all phases of the reproductive cycle, which means that chances to document potential male infanticidal behaviour was rather low throughout the observation nights. Therefore, it is impossible to assess the frequency of sexually selected infanticide in *L. silvicolum* with the data that is currently available.

Regardless of its frequency of occurrence, non-parental infanticide has severe fitness consequences for all individuals involved [3], [12]. Whereas consequences for the aggressor are usually positive, they are always negative for the victimized young and their respective mothers. Thus, infanticide may be an important cost of group living [62]. Infanticide is an evolutionary stable strategy in game-theoretic models [17]; its presence or absence may considerably influence the reproductive strategies of both sexes [3], [53], [63] and hence the respective social structure in any given species (reviewed in [1], [4], [64]).

As our study indicates, this influence might be applicable to bats as well. We are convinced that future studies, especially on polygynous, polyestrus bats in the tropics, will reveal more infanticidal species, making non-parental infanticide in general and sexually selected infanticide in particular a more widespread phenomenon in bats than previously thought [11]. The longevity and slow reproduction of chiropterans on the one hand [65] and their diverse social systems on the other hand, often governed by polygyny and a correspondingly high male reproductive skew [66], make bats a taxon prone to the evolution of sexually selected infanticide.

## Materials and Methods

This study was carried out in accordance with the ethical requirements of the University of Ulm and the American Society of Mammalogists (Animal Care and Use Committee; [67]). Field work was conducted on Barro Colorado Island (BCI), a field station belonging to the Smithsonian Tropical Research Institute, Panama. Our field work was approved by the Smithsonian Tropical Research Institute and complied with the laws and regulations of Panama. BCI is a 1,500 ha island located in Gatun Lake (9°09′N, 79°51′W) bordering the Panama Canal. A mosaic of young (ca. 100 years) and up to 600 years old semi-deciduous tropical lowland rainforest covers the island [68]. This forest supports abundant numbers of arboreal termite nests, in which *L. silvicolum* excavates roosting cavities [38], [40]. This species is a gleaning animalivorous bat [69] that occurs throughout lowland rainforests of Central and South America [70]. *Lophostoma silvicolum* belongs to the only genus of the family Phyllostomidae where all members exclusively roost in excavated live termite nests, mainly of *Nasutitermes corniger* [41].

We obtained data from a single roost. Every day, we conducted a visual census of the roosting bats without disturbing them by slowly walking up to the nest and by pointing a dimmed torch light into it. Throughout the study, the roost was occupied continuously (71 days) except for two consecutive days after an unsuccessful predation attempt by a snake (probably a small boa; determined by video analysis). During some nights, we observed social behaviors inside the termite nest by filming the roosting cavity with an infrared video camera (Dark Invader, 50 mm lens, F/1.3; B.E. Meyers Company, Redmond, WA, USA) installed on a tripod 1.7 m beneath the termite nest. The camera was either connected to a video camcorder (Canon ES 6000) or to a VHS recorder (Orion AC/DC). Illumination was provided by custom-built LED arrays. Video tapes were subsequently digitized and analysed using focal animal sampling [71].

We video-taped during 13 nights from early October to late November of 1998, which fell into the yearly rainy season lasting from May to December on BCI [72]. Heavy rain interrupted the video footage on five nights, whereas seven nights were filmed completely. Video recordings added up to 100 hours of observation time during which the roost was unoccupied for only 4.5 hours. During 68.5% of the overall recording time, more than two adult bats were present in the roost, during 20% at least one adult bat was present and during 7% one or two pups were roosting by themselves. The roost was occupied and maintained by one adult male, two adult females with one pup each, and sometimes up to four more adult females. The latter bats could not be individually identified but were assumed to be females since other studies report single male - multi female associations for *L. silvicolum* [42], [44]. One adult bat was sexed unequivocally as male, partly because his penis was clearly visible on some video recordings and partly because the bat's behavior was typical for a male (roost maintenance, copulation, position at the roost entrance; see [43] for details). The two lactating females could be distinguished from one another because one female was banded with a stainless steel necklace and the other one had a patch of bare skin on her back. The corresponding pups could be distinguished based on their respective body size: the pup of the banded female was younger and therefore somewhat smaller than the pup of the other female. The sex of the pups was unknown. Both pups were not volant at the beginning of the observation period.

Social behaviors were described in detail and summarized in an ethogram (Table 1). We watched the video footage in real time and noted the duration of every state and the occurrence of every event (*sensu* [71]) for every bat present in the roost. For clarity, we produced sketches of selected behaviors that were obtained from the video recordings. All statistical tests were performed using SPSS version 17.0 (SPSS, Chicago, IL, USA). Sequential Bonferroni corrections were applied following [73].
